# Same but different. Incidental and symptomatic lower grade gliomas show differences in molecular features and survival

**DOI:** 10.1007/s11060-023-04301-x

**Published:** 2023-04-12

**Authors:** Matthias Demetz, Aleksandrs Krigers, Patrizia Moser, Johannes Kerschbaumer, Claudius Thomé, Christian F. Freyschlag

**Affiliations:** 1 Department of Neurosurgery, Medical University of Innsbruck, Anichstr. 35, Innsbruck, AT-6020 Austria; 2 Department of Neuropathology, University Hospital Innsbruck, Tirol Kliniken, Innsbruck, Austria

**Keywords:** LGG, Incidental gliomas, Overall survival, Neuro-oncology

## Abstract

**Purpose:**

Data on differences in overall survival and molecular characteristics between incidental (iLGG) and symptomatic lower grade Glioma (sLGG) are limited. The aim of this study was to investigate differences between patients with iLGG and sLGG.

**Methods:**

All adult patients with a histologically proven diffuse (WHO°II) or anaplastic (WHO°III) glioma who underwent their first surgery at the authors’ institution between 2010 and 2019 were retrospectively included. Tumor volume on pre- and postoperative MRI scans was determined. Clinical and routine neuropathological data were gained from patients’ charts. If IDH1, ATRX and EGFR were not routinely assessed, they were re-determined.

**Results:**

Out of 161 patients included, 23 (14%) were diagnosed as incidental findings. Main reasons for obtaining MRI were: headache(n = 12), trauma(n = 2), MRI indicated by other departments(n = 7), staging examination for cancer(n = 1), volunteering for MRI sequence testing(n = 1). The asymptomatic patients were significantly younger with a median age of 38 years (IqR28-48) vs. 50 years (IqR38-61), p = 0.011. Incidental LGG showed significantly lower preoperative tumor volumes in T1 CE (p = 0.008), FLAIR (p = 0.038) and DWI (p = 0.028). Incidental LGG demonstrated significantly lower incidence of anaplasia (p = 0.004) and lower expression of MIB-1 (p = 0.008) compared to sLGG. IDH1-mutation was significantly more common in iLGG (p = 0.024). Incidental LGG showed a significantly longer OS (mean 212 vs. 70 months, p = 0.005) and PFS (mean 201 vs. 61 months, p = 0.001) compared to sLGG.

**Conclusion:**

Our study is the first to depict a significant difference in molecular characteristics between iLGG and sLGG. The findings of this study confirmed and extended the results of previous studies showing a better outcome and more favorable radiological, volumetric and neuropathological features of iLGG.

## Introduction

With easier access to imaging techniques, especially magnetic resonance imaging (MRI), the occurrence of incidentally detected tumors including gliomas has increased in recent years [1, 2]. Incidental diffuse and anaplastic gliomas (iLGG) are characterized by slow tumor growth with patients staying asymptomatic for a long time [3]. While in symptomatic diffuse and anaplastic gliomas (sLGG) treatment clearly favors early surgical resection [4], the evidence in iLGG is still debatable. Historically, wait-and-see strategy has often been chosen in case of iLGG – until the patients became symptomatic or signs of malignant transformation like contrast enhancement in MRI were detected [5]. On the other hand, recent data showed a prognostic advantage of early resection of iLGG even at an asymptomatic stage [6].

Many factors influence outcome and overall survival (OS) in gliomas like extent of resection (EOR) [7, 8] and anaplasia [9]. With tremendous advances in molecular sciences, specific genetic alterations could be associated with changes in patients’ prognosis. Isocitrate dehydrogenase 1 (IDH1)-mutated tumors demonstrated a significantly better outcome compared to IDH1-wildtype gliomas in many studies [10–13]. Furthermore, with the updated World Health Organization (WHO) classification of 2021 the importance of molecular features increased [14].

Despite advances in the understanding of the clinical course of iLGG, no differences in molecular characteristics between iLGG and sLGG have been shown. In addition, literature on differences in OS and progression free survival (PFS) between sLGG and iLGG is limited.

The aim of this study was therefore to investigate epidemiological, radiological and neuropathological differences between patients with iLGG and sLGG as well as to compare the OS and PFS between these two groups in order to gain a better understanding of the pathogenesis and clinical course of iLGG.

## Materials & methods

In this study, all adult patients (≥ 18 years at the time of surgery) who underwent first surgery (resection or biopsy) on a diffuse (WHO °II) or anaplastic (WHO °III) cerebral glioma according to the WHO classification of 2016 between 2010 and 2019 at the authors’ institution were retrospectively included. Pre-treated patients were excluded. Patients with extracranial tumor location were also excluded.

Epidemiological, clinical and follow-up data could be gained from the institutional neuro-oncological database. Patients’ preoperative symptoms and reasons for undergoing imaging were gathered from patient records and documented in detail during the admission interview. All patients whose imaging or signs/symptoms could not be explained by a local or systemic effect of the glioma were considered as incidental findings (iLGG cohort). This includes cranial imaging after trauma or imaging after diagnosis obtained for other diagnoses (e.g. ENT). All patients with preoperative tumor-related symptoms were identified as sLGG.

Surgical resection was performed as standard of care at the authors’ institution in all eligible patients. In case of a non-resectable glioma location (like brain stem or thalamus), only biopsy and radio-chemotherapy[15, 16] was performed. Patients were followed postoperatively at three- or six-monthly intervals, depending on the WHO grade and molecular characteristics of the LGG. Tumor progression was defined based on the Response assessment in neuro-oncology (RANO) criteria [17]. Additional surgical procedures for postoperative complications like bleeding, CSF fistula or wound healing problems until last follow-up were gained from patients’ charts. Patients’ general condition was assessed according to the Eastern Cooperative Oncology Group (ECOG) pre- and postoperatively.

Neuropathological assessment was routinely performed on FFP-embedded tissue by a team of experienced neuropathologists and the histological diagnosis was based on the revised 4th WHO grading system of central nervous system tumors of 2016 [18, 19]. IDH1-mutation in the R132H position was tested with immunohistochemistry (IHC) and in case of a negative result we performed DNA sequencing for patients under 40 years to approve the wildtype IDH1 and IDH2 status. Expression of nuclear alpha thalassemia mental retardation X-linked (ATRX), epidermal growth factor receptor (EGFR) and MIB-1 as proliferation marker were assessed with IHC. If IDH, ATRX and EGFR were not assessed routinely and tissue was available for further analysis, the parameters were re-determined. There were some tumors where no tissue was available.

According to individual neuropathological diagnosis, each patient was individually discussed in the institutional multidisciplinary tumor board to decide on adjuvant treatment, following international guidelines [20–23]. Patients with higher risk tumors were assigned to adjuvant therapy: in case of anaplasia, incomplete resection, wild-type IDH1 or lost nuclear ATRX. Lower risk tumors (complete resection, IDH1-mutation, no anaplasia) were observed. Following international guidelines and after the discussion in the institutional multidisciplinary tumor board high risk patients (anaplasia, IDH-wt, incomplete resection) were followed-up with 3 months intervals, patients with lower risk were followed-up in 6 months intervals.

**Fig. 1:**
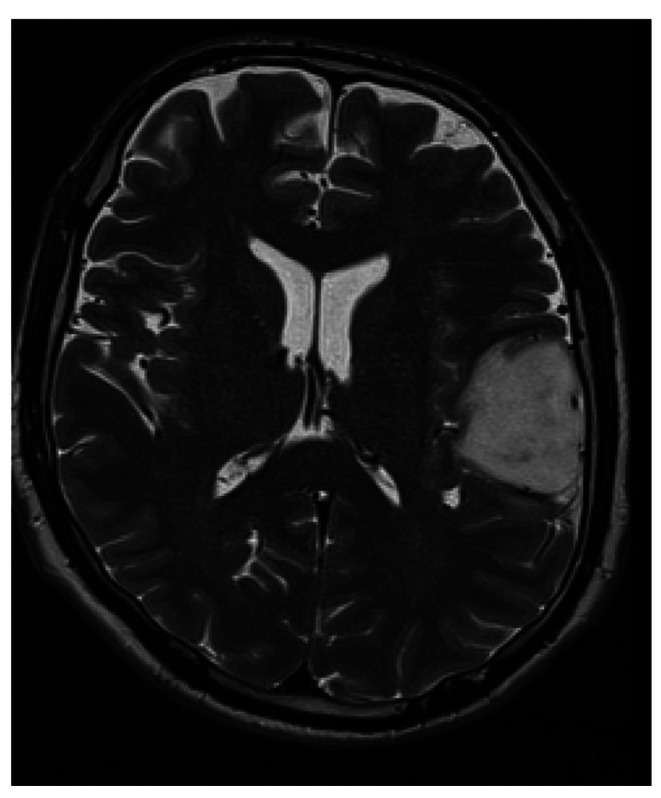
LGG before volumetric segmentation in T2 weighted MRI.

**Fig. 2:**
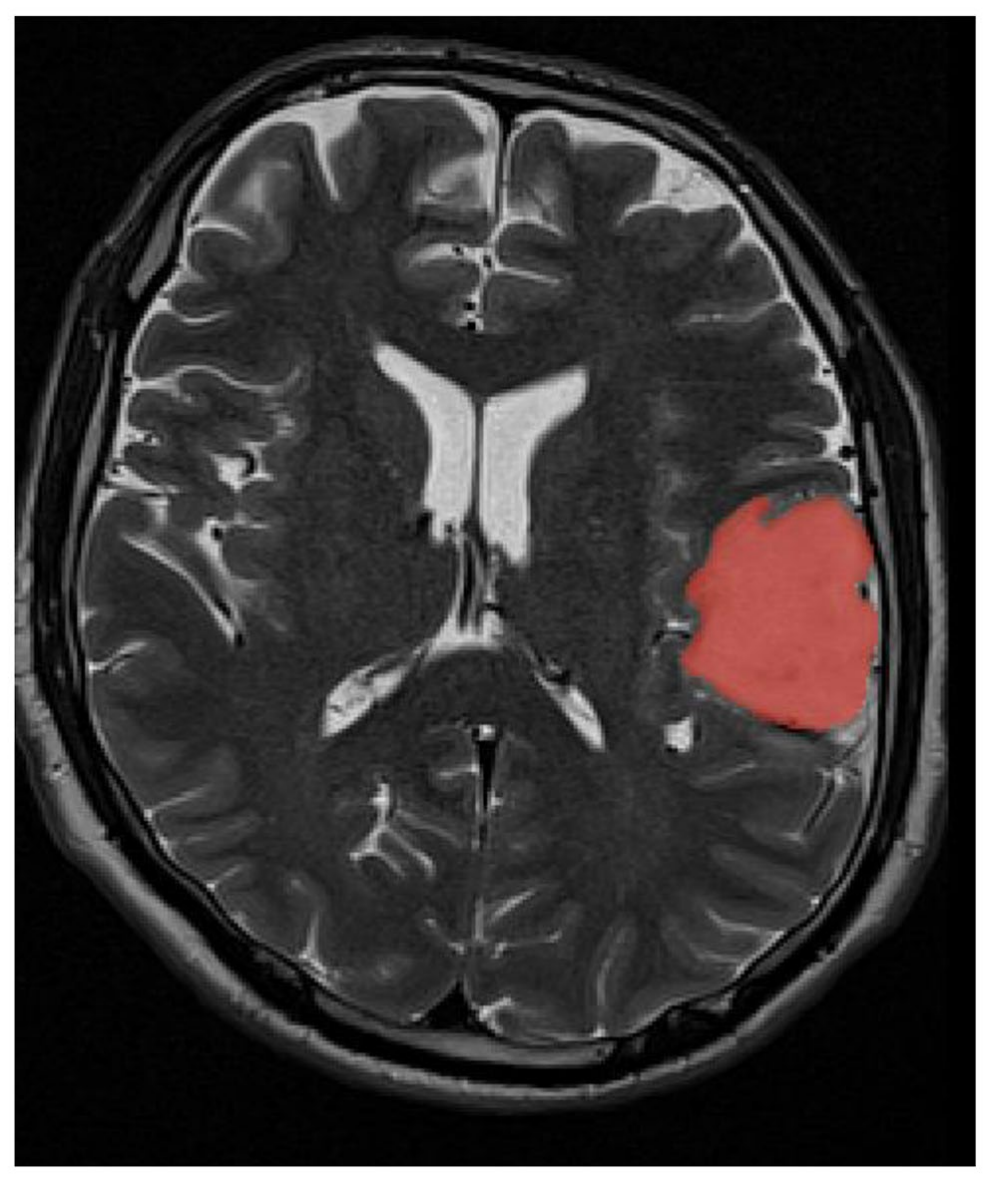
LGG after volumetric segmentation in T2 weighted MRI.

MRI including T1-weighted Gadolinum-contrasted as well as native T1, T2, FLAIR and DWI sequences was performed preoperatively and postoperatively within 72 h as the standard of care for patients harboring LGG [24]. Tumor volume was manually measured using segmentation in ITK-SNAP software in T1 CE as well as native T1, T2, FLAIR (fluid attenuated inversion recovery) and DWI (diffusion weighted imaging) sequences (v.3.8.0 for Mac OS, UPenn and UNC dev.), as shown in Figs. 1 and 2.

Data evaluation was performed with IBM SPSS Statistics (IBM SPSS Statistics for Mac OS, Version 27.0. Armonk, NY: IBM Corp.). Scale variables were assessed with T-tests and shown as mean with standard deviation (SD) in case of normal distribution or with Mann-Whitney U-test and demonstrated as median with interquartile range (IqR) if normal distribution was not achieved. Binominal pairs were compared with Chi-squared test. The mean estimated PFS and OS times were calculated with Kaplan–Meier processing and analyzed with LogRank test. Cox regression assessment was used to reveal hazard ratios (HR) for oncological progression or death. The α value was 0.05, and 95% confidence intervals were constructed.

This study was approved by the ethics committee of Medical University of Innsbruck (1333/2021) and conducted in accordance with the ethical standards as laid down in the 1964 Declaration of Helsinki and its later amendments.

## Results

161 patients (69 female, 92 male) with a median age of 47 years at the time of surgery (IqR 36–58, absolute range 18–86) met the inclusion criteria. From these, 23 cases (14%, 12 female and 11 male) were identified as incidental findings. The reasons for the first-time imaging in the incidental group were headache and migraine in 12 cases, imaging for other medical departments (ENT, ophthalmology) in 7 cases, trauma in 2 cases, CT-staging examination after breast cancer in 1 case and volunteering for MRI device testing in 1 case.

20 patients with iLGG and 101 with sLGG underwent resection, while in 3 iLGG and 37 sLGG cases only biopsy was performed due to tumor location or patients’ decision.

We could not find any significant differences regarding gender (p–n.s.). In the asymptomatic group the authors assessed 12 female and 11 male patients, while in the symptomatic group there were 57 female and 81 male patients.

Patients with iLGG were significantly younger in this study with a median age of 38 years (IqR 28–48) compared to a median of 50 years (IqR 38–61) in patients with sLGG, p = 0.011. Furthermore, iLGG presented with a significantly better performance status according to the ECOG with a mean preoperative score of 0.09 (SD 0.29) versus mean 0.54 (SD 0.73), p = 0.002. Incidental LGG demonstrated better postoperative ECOG as well compared to sLGG with mean 0.13 (SD 0.34) versus 0.55 (SD 0.73), p = 0.004.

Most frequent tumor location for both iLGG and sLGG was the frontal lobe, 12/23 (52%) and 75/138 (54%) cases correspondingly. Temporal tumor location (55/138 (40%) vs. 2/23 (9%)) was found significantly more frequently in sLGG (p = 0.004), while iLGG presented more likely at the cerebellum (3/23 (13%) vs. 4/138 (3%), p = 0.027). Symptomatic LGG trended to be more frequent left-sided than iLGG (72/138 (52%) vs. 7/23 (30%), p = 0.064).

Differences in molecular and neuropathological features can be found in Table 1. Tumor volumes before and after surgery of symptomatic and asymptomatic LGG can be found in Table 2. Incidental LGG showed significantly lower tumor volumes in various MRI sequences. Both iLGG and sLGG could be resected successfully, as shown by the results in Table 2. However, considering the significantly lower tumor volume preoperatively in iLGG, a less invasive surgery can be assumed for iLGG to achieve a comparable result. Regarding the extent of resection (EOR) in percent, iLGG showed significantly higher EOR compared to sLGG in both T2 (p = 0.044) and DWI (p = 0.039), as shown in Table 3.

**Table 1:** Differences in neuropathological features are showed for iLGG and sLGG. Patients with incidental tumors showed significantly more often a favorable IDH1 mutation and a significant lower probability of anaplasia as well as lower proliferation rates. No significant differences could be proven for the expression of ATRX and EGFR.

	sLGG	iLGG	P-value
Diffuse vs. anaplastic	49 (36%) vs. 89 (64%)	16 (70%) vs. 7 (30%)	**0.002**
Median MIB-1 (IqR)	16.0% (4.5–27.5)	10.0% (3.5–16.5)	**0.008**
IDH1 mutated vs. wild-type	68 (56%) vs. 53 (44%), 17 missing	18 (82%) vs. 4 (18%), 1 missing	**0.024**
ATRX loss vs. expression	36 (31%) vs. 80 (69%), 22 missing	8 (40%) vs. 12 (60%), 3 missing	0.429
EGFR overexpression vs. no overexpression	84 (71%) vs. 34 (29%), 20 missing	13 (65%) vs. 7 (35%), 3 missing	0.576

**Table 2:** Differences in mean preoperative and postoperative tumor volumes in different MRI sequences are shown for iLGG and sLGG. Incidental LGG showed significantly lower tumor volumes in various MRI sequences. Postoperative tumor volumes are shown for both resected and biopsied patients

Tumor volume	sLGG (SD)	iLGG (SD)	P-value
T2 preoperative	44.5 cm^3^ (44.3)	29.9 cm^3^ (33.1)	0.06
T1 CE preoperative	4.7 cm^3^ (10.7)	0.8 cm^3^ (2.2)	**0.008**
FLAIR preoperative	59.9 cm^3^ (56.1)	38.5 cm^3^ (38.9)	**0.038**
DWI preoperative	56.1 cm^3^ (54.5)	34.3 cm^3^ (38.1)	**0.028**
T2 postoperative	7.8 cm^3^ (17.8)	5.3 cm^3^ (13.9)	0.136
T1 CE postoperative	0.3 cm^3^ (1.7)	0.5 cm^3^ (2.2)	0.238
FLAIR postoperative	12.8 cm^3^ (29.2)	8.4 cm^3^ (22.1)	0.159
DWI postoperative	9.9 cm^3^ (23.4)	4.9 cm^3^ (12.3)	0.120

**Table 3:** Differences in mean EOR are shown for iLGG and sLGG in all resected cases. ILGG showed significantly higher EOR in T2 and DWI as well as a strong trend in FLAIR. Due to the not always CE tumors in LGG, no significant differences could be found in T1 CE.

Extent of resection	sLGG (SD)	iLGG (SD)	P-value
T2	84.7% (27.8%)	94.9% (10.9%)	**0.044**
T1 CE	93.9% (18.4%)	100% (0%)	0.231
FLAIR	82.8% (28.4%)	94.1% (12.8%)	0.067
DWI	84.4% (27.1%)	96.5% (10.4%)	**0.039**

We could not find any significant differences regarding additional surgical procedures due to postoperative complications between the two groups. 10 out of 138 (7.2%) sLGG vs. 1 out of 23 (4.3%) iLGG required further surgical treatments within 30 days surgery (p–n.s.). Reasons for second surgery were wound healing in 3 cases, wound infections in 4 cases, CSF fistula in 1 case and space-occupying bleeding in 2 cases in the sLGG cohohrt, while 1 case of wound infection occurred in the iLGG cohort. Despite the complications, iLGG showed no permanent neurological deficits.

103 out of 180 patients (57%) with sLGG received adjuvant treatment including radiotherapy and temozolamid. In the iLGG cohort 11 out of 26 patients (42%) were treated accordingly.

Patients with symptoms demonstrated both a significant shorter estimated PFS (mean 60.9 months (SD 5.8) vs. 201.5 months (SD 22.9), p = 0.001) and estimated OS (mean 70.4 months (SD 5.2) vs. 212.4 months (SD 15.3), p = 0.005) than asymptomatic patients, as demonstrated in the following Kaplan-Meier-curves. Cox regression revealed a Hazard Ratio (HR) of 0.129 (Confidence Interval 0.031–0.530) for shorter PFS and of 0.168 (Confidence interval 0.041–0.685) for shorter OS for iLGG, resulting in an almost 7 times higher risk of symptomatic patients for an earlier progression and an almost 5-fold higher risk for decreased OS as compared to primarily asymptomatic patients.

**Fig. 3:**
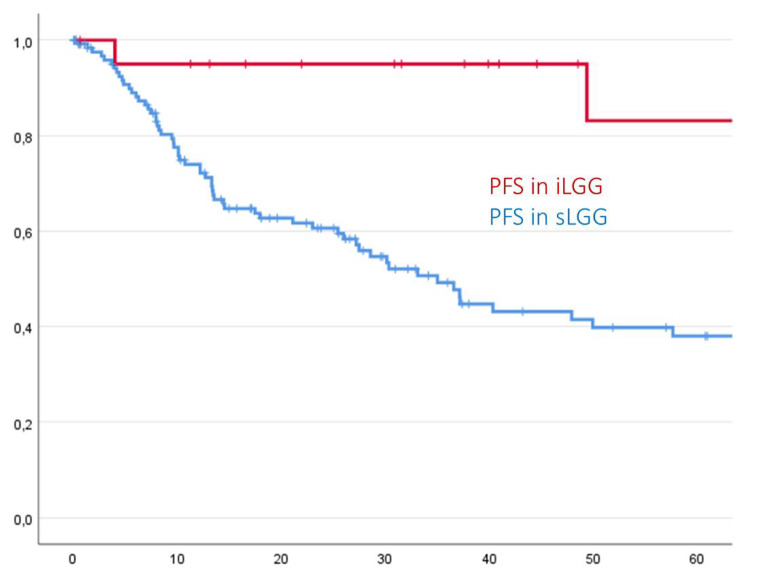
Incidental LGG showed a significantly longer PFS compared to sLGG in Kaplan-Meier analysis (p < 0.001)

**Fig. 4:**
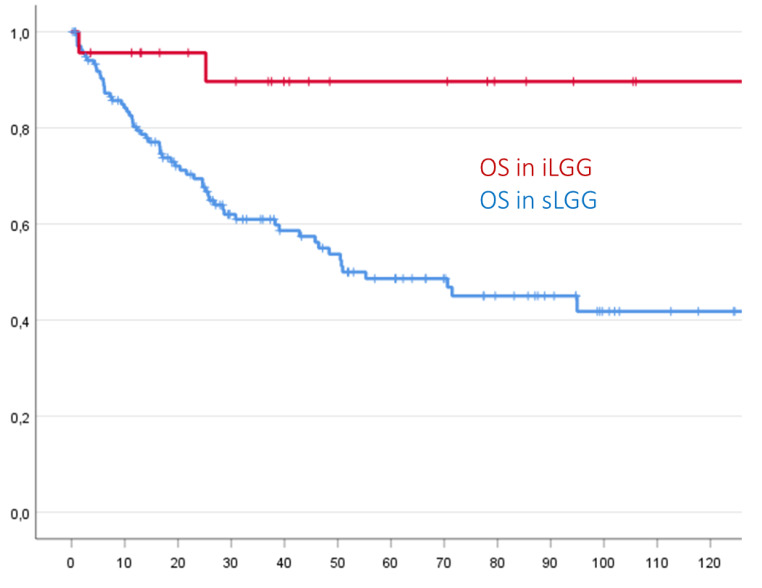
Incidental LGG showed a significantly longer OS compared to sLGG in Kaplan-Meier analysis (p = 0.005)

Differences between iLGG and sLGG regarding PFS and OS are shown in Figs. 3 and 4.

## Discussion

We demonstrated significantly more favorable neuropathological characteristics, such as IDH1-mutation, lack of anaplasia and lower mitotic rates in incidental diffuse and anaplastic gliomas compared to a group of symptomatic gliomas. Additionally, previously described characteristics for iLGG such as younger age, better ECOG performance, smaller preoperative tumor volumes, higher EOR and better PFS and OS were confirmed.

Incidental gliomas pose a challenge to treating physicians because of their rarity and lack of clear guidelines. This study identified 14% of all cases to be incidental, which is consistent with previous findings [25–30]. Enabling easy access to imaging, especially MRI, the number of incidental tumors is expected to further increase, which underpins the importance of studies on incidental gliomas to gain a better understanding for the best possible treatment option.

The significantly younger age at the time of surgery as well as the significantly lower preoperative tumor volumes of iLGG in this study could be related to the fact that tumor had less time to grow and therefore did not reach the significant volume to cause symptoms, suggesting that iLGG may represent the earlier stage of sLGG as this has been hypothesized before.[31, 32].

Furthermore, a lower preoperative tumor volume enhances the probability of a gross total resection (GTR) of iLGG. Further, better postoperative performance scores and low risk of permanent neurological deficits in iLGG have been shown in previous studies [6, 27, 29]. A “wait and see” (“wait and scan”) strategy allows the tumor to enlarge longitudinally, as this has been described by Pallud et al. [31, 32]. A larger volume harbors an increased likelihood of symptoms combined with a lesser possibility of achieving GTR, which is a strong independent predictor for OS in patients harboring gliomas [33–39].

Incidental LGG demonstrated a significantly better ECOG performance score both pre- and postoperatively compared with sLGG, which could be expected since the first group was neurologically asymptomatic and younger. The postoperatively better ECOG performance score demonstrates that surgical treatment can be performed safely particularly in asymptomatic patients with no higher risk of postoperative complications or permanent neurological deficits [40, 41].

OS is not only determined by the EOR but also by molecular and neuropathological features of LGG. In this study, the authors found significantly more favorable molecular and neuropathological characteristics in iLGG compared to sLGG. Patients with iLGG were more likely to have an IDH1 mutation which translates towards increased OS. At the same time, we found a significantly lower risk of anaplasia in iLGG (only 30% in iLGG vs. 64% in sLGG), which suggests that gliomas with unfavorable molecular features (like IDH1-wt) grow more quickly, become therefore symptomatic and included partial malignant transformation at an earlier time in the course of the disease.

With regard to ATRX and EGFR alterations, no significant differences between iLGG and sLGG could be discovered. These two molecular markers, particularly important in diagnosis of astrocytic tumors and glioblastomas, appear to play a minor role in incidental gliomas in our cohort. However, further multicenter studies will be needed to better understand their impact on symptoms in gliomas.

Favorable molecular, neuropathological, epidemiological, radiological and surgical characteristics lead to a significantly better PFS and OS in patients with iLGG. We demonstrated an almost 7 times higher hazard for earlier progression and an almost 5 times higher hazard for shorter OS in patients with sLGG compared to patients with iLGG. This could be of clinical relevance, helping the treating physicians to explain the patients the benefits of a treatment even at an asymptomatic stage. The better PFS and OS mandate not only for early maximum safe resection in iLGG, but also strongly argues against a wait and see (wait and scan) strategy, which was historically justified by the generally slow growth of LGG and the risk of postoperative deficits [6, 26, 42]. Considering the slow but steady growth of LGG and increasing risk for symptoms and malignant transformation, it can be assumed that a favorable course is more likely in case of early surgery compared to a wait and see strategy.[31, 32, 43] Considering the recent literature, the difficulty of asymptomatic patients especially at a very young age becomes evident. The authors described their concern about a potential neurological deterioration after surgery in case of a young mother with an asymptomatic glioma. However, after surgery she showed no new neurological deficits, what is in accordance with our findings [44].

Considering neurosurgical studies on supramarginal resection in glioma surgery, this could lead to longer survival in iLGG. Previous studies suggested a higher incidence of EOR as well as supramarginal resection in iLGG because of the lower tumor volume and lower risk of eloquent tumor location [45]. With an increasing number of studies who suggest a more aggressive surgical strategy aiming to resect the peri-tumoral infiltrated areas as well, iLGG could benefit from their lower incidence of non-eloquent tumor location, since the higher EOR could also been translated into a longer PFS and OS [46, 47]. Furthermore, considering the higher OS in case of a supramarginal resection reported from literature, this again favors an early resection of iLGG before an infiltration in eloquent areas occurs. However, supramarginal resection was not mandatory in our series and cannot be evaluated [48, 49].

Our study has limitations. This was a retrospective single-center study. The PFS and especially the OS were not reached by all patients because of the low malignant course of especially IDH1 mutated gliomas. We had no access to a wait-and-see cohort, since the department standard following international studies preferred an early surgical treatment of both iLGG and sLGG. There might be a potential bias in case of very deep seated iLGG or iLGG with eloquent location. Especially in young asymptomatic patients with a glioma not feasible for resection, biopsy might not have always been performed. These patients have not been included in our analysis, since only histologically confirmation has been a crucial inclusion criterion. Due to the routinely assessed ECOG performance score we included the ECOG Score and not the Karnofsky Performance Score. However, previous oncological studies showed comparable results for both performance scores in various tumor diagnosis [50, 51]. Due to the retrospective study design and limited tissue availability, key molecular markers could not be determined in all patients. Therefore, a retrospective stratification according to the updated WHO classification of 2021 was not entirely possible. Further prospective multicenter studies will be needed to deepen the knowledge of molecular characteristics that gained importance with the updated WHO classification of 2021 and their impact on the clinical course of iLGG.

## Conclusion

Incidental LGG were associated with favorable prognostic features like lower mitotic activity, lack of anaplasia and presence of IDH1 mutation. These patients had better pre- and postoperative performance. The probability of total resection without causing permanent neurological deficits was high. Consequently, patients with iLGG compared to sLGG showed 7 times more favorable PFS and 5 times more favorable OS hazards.

## Data Availability

The datasets generated during and/or analyzed during the current study are available from the corresponding author on reasonable request.
